# Cycling and walking to work in New Zealand, 1991-2006: regional and individual differences, and pointers to effective interventions

**DOI:** 10.1186/1479-5868-6-64

**Published:** 2009-09-20

**Authors:** Sandar Tin Tin, Alistair Woodward, Simon Thornley, Shanthi Ameratunga

**Affiliations:** 1 Section of Epidemiology and Biostatistics, School of Population Health, University of Auckland, Private Bag 92019, Auckland 1142, New Zealand; 2 School of Population Health, University of Auckland, Private Bag 92019, Auckland 1142, New Zealand

## Abstract

**Background:**

Active commuting increases levels of physical activity and is more likely to be adopted and sustained than exercise programmes. Despite the potential health, environmental, social and economic benefits, cycling and walking are increasingly marginal modes of transport in many countries. This paper investigated regional and individual differences in cycling and walking to work in New Zealand over the 15-year period (1991-2006).

**Methods:**

New Zealand Census data (collected every five years) were accessed to analyse self-reported information on the "main means of travel to work" from individuals aged 15 years and over who are usually resident and employed in New Zealand. This analysis investigated differences in patterns of active commuting to work stratified by region, age, gender and personal income.

**Results:**

In 2006, over four-fifths of New Zealanders used a private vehicle, one in fourteen walked and one in forty cycled to work. Increased car use from 1991 to 2006 occurred at the expense of active means of travel as trends in public transport use remained unchanged during that period. Of the 16 regions defined at meshblock and area unit level, Auckland had the lowest prevalence of cycling and walking. In contrast to other regions, walking to work increased in Wellington and Nelson, two regions which have made substantial investments in local infrastructure to promote active transport. Nationally, cycling prevalence declined with age whereas a U-shaped trend was observed for walking. The numbers of younger people cycling to work and older people walking to work declined substantially from 1991 to 2006. Higher proportions of men compared with women cycled to work. The opposite was true for walking with an increasing trend observed in women aged under 30 years. Walking to work was less prevalent among people with higher income.

**Conclusion:**

We observed a steady decline in cycling and walking to work from 1991 to 2006, with two regional exceptions. This together with the important differences in travel patterns by age, gender and personal income highlights opportunities to target and modify transport policies in order to promote active commuting.

## Background

Physical activity provides substantial health benefits such as avoiding premature deaths [1], lowering the risk of a range of health conditions, notably cardiovascular diseases [2] and some forms of cancer [3], and enhancing emotional health [4]. While regular physical activity (i.e., undertaking at least 30 minutes of moderate intensity physical activity on most, if not all, days of the week) is recommended to promote and maintain health [5-7], maintenance of such activity has been identified as a major barrier for health behaviour interventions [8,9]. Previous research suggests that active commuting (building cycling and walking into daily life) may be more likely to be adopted and sustained compared with exercise programmes [10].

We have found published evidence of a variety of health benefits associated with active commuting. For example, obesity rates are lower in countries where active travel is more common [11]. A recent review reported that active commuting was associated with an 11% reduction in cardiovascular event rates [12]. A Copenhagen study found a 28% lower risk of mortality among those who cycled to work, even after adjusting for leisure time physical activity [13]. Similar associations were observed among Chinese women who cycled or walked for transportation [14]. In addition, active commuting may enhance social cohesion, community livability and transport equity [15-17], improve safety to all road users [18], save fuel and reduce motor vehicle emissions. A previous study predicted that if recommended daily exercise was swapped for transportation, this could reduce 38% of US oil consumption (for walking and cycling) and 11.9% of US's 1990 net emissions (for cycling), and could burn 12.2 kg of fat per person annually (for walking) and 26.0 kg of fat per person annually (for cycling) [19].

These effects are important not only in high-income countries in which the private motor vehicle has long been the dominant mode of transport but also in rapidly industrialising parts of the world, such as China, in which active commuting was until recently very common, but is now being replaced by motorised transport [20].

New Zealand is among the countries with the highest rate of car ownership in the world (607 cars per 1000 population) [21]. Driver or passenger trips account for four-fifths of the overall travel modal share [22] although one third of vehicle trips are less than two kilometres and two-thirds are less than six kilometres [23]. While the national Transport Strategy aims to "increase walking and cycling and other active modes to 30% of total trips in urban areas by 2040" [24], this target is unlikely to be met given current patterns of expenditure on the transport network [25].

Travel to work makes up about 15% of all travel in New Zealand [22]. Use of private motor vehicles is the dominant mode of travel to work [26] and may be sensitive to changing oil price [27]. The aim of this study was to investigate regional and individual differences in cycling and walking to work in the employed Census population over the 15-year period between 1991 and 2006. Possible intervention and policy options to promote active commuting will be discussed from New Zealand and international perspectives.

## Methods

This paper presents an analysis of aggregate data obtained from the New Zealand Census undertaken by Statistics New Zealand every five years. Each Census since 1976 has collected information about the "main means of travel to work". However, the question was not date-specific prior to 1991.

The last four Censuses (1991, 1996, 2001 and 2006) asked usually resident employed persons aged 15 years and over about their main mode of transport to work on the date of Census (first Tuesday in March). For example, the 2006 Census asked the question "On Tuesday 7 March what was the one main way you travelled to work - that is, the one you used for the greatest distance?" and response options included: worked at home; did not go to work; public bus; train; drove a private car, truck or van; drove a company car, truck or van; passenger in a car, truck, van or company bus; motorbike; bicycle; walked or jogged; and other. The non-response rates to this particular question were 1.6%, 3.3%, 3.5% and 3.7% for the 1991, 1996, 2001 and 2006 Census respectively. The sample for this study was restricted to those who travelled to work on the specified day (i.e., those who reported "worked at home" or "did not go to work" were excluded, which ranged from 18% in 1991 to 22% in 2001).

The 'means of travel to work' responses were categorised into four main groups: "bicycle", "walk", "public transport" (including "public bus" and "train" responses) and "vehicle driver/passenger" (including "drove a private car, truck or van", "drove a company car, truck or van" and "passenger in a car, truck, van or company bus" responses). Trends in the main means of travel to work were presented for the 30-year period (1976 to 2006). As the data collected prior to 1991 were not date specific, the 1991 and 2006 Census data were used to examine trends in cycling and walking to work by region, age and gender. There are a total of 16 regions in New Zealand defined at meshblock and area unit levels: nine in the North Island and seven in the South Island. A meshblock is the smallest geographic area containing an average of 100 people and 40 dwellings [28]. Total personal income before tax in the 12 months ending 31 March was collected as a range and the data were analysed for the 2006 Census only due to limited comparability of data across Censuses. All data were self-reported and only aggregate data were available for this analysis.

The Ministry of Transport's Household Travel Survey data (2003-2008) [29] were used to compute the average distance of home to work trips in each region. It is a national survey collecting data on personal travel from about 3500 people (from about 2000 households) throughout New Zealand each year. The data were weighted to account for household and person non-responses. Information on other regional characteristics was obtained from the Statistics New Zealand (population density) [30] and the National Institute of Water & Atmospheric Research (climate status) [31]. The relationship between these characteristics and participation levels of active transport were measured using Spearman's rank correlation coefficient and linear and non-linear regression.

## Results

**Figure 1:**
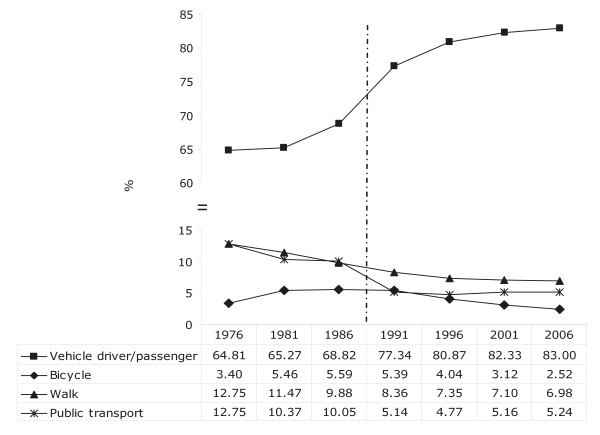
**Mode of travel to work on the census day in the usually resident employed population aged 15 years and over (1976 to 2006)**.

The majority of people travelled to work by car, with an increasing trend over time from 64.8% in 1976 to 83.0% in 2006 (Figure 1). In contrast, walking to work declined over this 30 year period (12.8% in 1976 to 7.0% in 2006). The prevalence of cycling to work increased slightly from 1976 (3.4%) to 1986 (5.6%) and then declined steadily. In 2006, only 2.5% of people who travelled to work used a bicycle. The prevalence of public transport use decreased from 12.8% in 1976 to 5.1% in 1991 but remained stable at around 5.0% over the last 15 year period.

### Regional differences in cycling and walking to work

Regional variation in active transport along with environmental and geographic factors thought to influence this variation is presented in Table 1. Auckland is the most populated region and West Coast, the least. The average distance of the trip to work varies from 6.7 km in West Coast to 14.8 km in Waikato. There is a moderate variation in average temperatures and sunshine hours with highest levels recorded in regions in the north of the South Island; and a three-fold variation in rainfall across the major urban areas of different regions around the time of the census.

**Table 1:** Regional characteristics and correlations with the prevalence of cycling and walking to work

**Region**	**Population density (per km^2^)^1^** **2006**	**Average distance of home-work trips (km)^2 ^(95% CI) ** **2003-2008**	**Average sunshine (hours)^3^** **1971-2000**	**Average rainfall (mm)^3^** **1971-2000**	**Average air temperature (°C)^3^** **1971-2000**
Northland	10.8	12.2 (7.0-17.4)	153	144	18.6
Auckland	215.3	10.9 (9.9-12.0)	180	82	18.7
Waikato	15.9	14.8 (11.1-18.5)	184	87	17.1
Bay of Plenty	21.0	9.5 (6.8-12.1)	197	132	18.3
Gisborne	5.3	8.3 (5.2-11.5)	185	99	17.4
Hawke's Bay	10.5	9.2 (6.4-12.1)	194	85	17.7
Taranaki	14.3	9.3 (5.1-13.6)	202	108	16.9
Manawatu-Wanganui	10.0	9.5 (7.4-11.6)	170	74	16.6
Wellington	55.2	12.4 (10.2-14.6)	191	92	16.6
Tasman	4.6	8.7 (6.4-11.1)*	212	75	16.3
Nelson	96.8	8.7 (6.4-11.1)*	212	77	16.1
Marlborough	3.9	8.7 (6.4-11.1)*	224	54	16.3
West Coast	1.3	6.7 (5.5-7.9)	161	171	15.7
Canterbury	11.7	10.1 (7.6-12.6)	183	56	15.1
Otago	6.2	9.3 (6.1-12.6)	139	70	13.7
Southland	2.8	9.9 (6.6-13.3)	136	94	12.5

***Spearman Correlation Coefficient (p-value)***					
% cycling to work (2006)	-0.25 (0.4)	-0.64 (0.007)	0.58 (0.02)	-0.46 (0.07)	-0.36 (0.2)
% walking to work (2006)	-0.29 (0.3)	-0.27 (0.3)	-0.03 (0.9)	-0.15 (0.6)	-0.62 (0.01)

1 -- Source: Indicator 2: Living density. Statistics New Zealand

2 -- Source: Household Travel Survey data. Ministry of Transport

3 -- Historical averages for the main cities/centres in March. Source: NIWA National Climate Database. National Institute of Water & Atmospheric Research

* - Average distance for three regions

**Figure 2:**
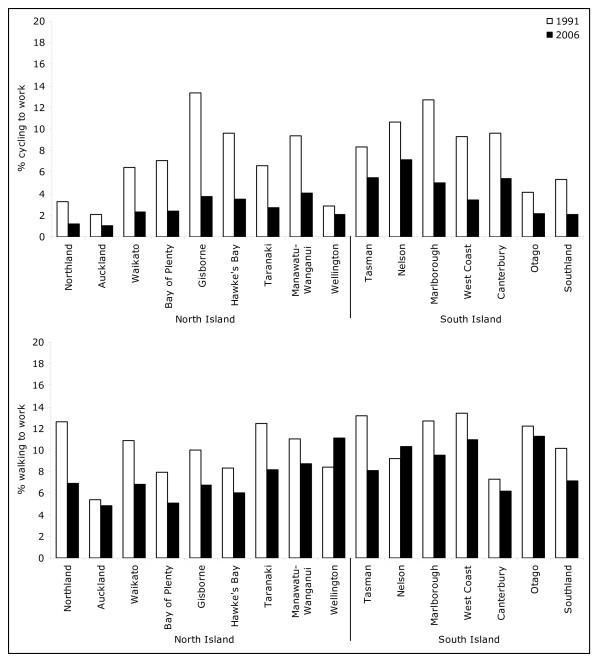
**Proportion of people who cycled and walked to work on the census day by area of usual residence (1991 to 2006)**.

Active travel to work varied widely across regions. In 2006, Nelson had the highest prevalence of cycling (7.2%) and Auckland, the lowest (1.0%) (Figure 2). All regions experienced a sharp fall in cycling prevalence, most steeply in Gisborne, over the 15 year period between 1991 and 2006. Walking prevalence was highest in Otago (11.3%), Wellington (11.1%) and West Coast (10.9%) and lowest in Auckland (4.9%). Contrary to other regional trends, the proportion of people who walked to work in Wellington and Nelson increased from 1991 to 2006.

**Figure 3:**
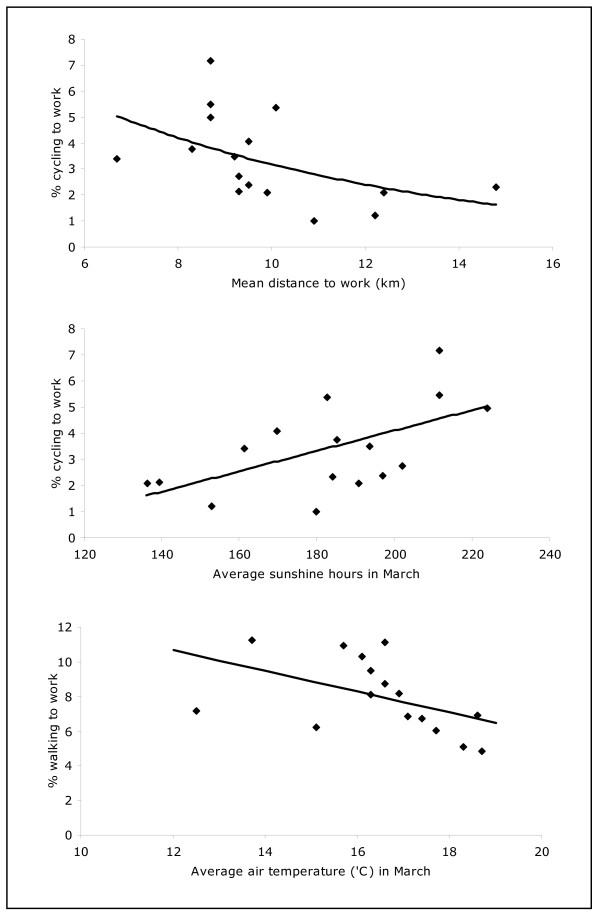
**Relationship between the prevalence of cycling and walking to work and specific regional factors**.

The prevalence of cycling to work was negatively correlated with the average distance of home to work trips and positively correlated with average sunshine hours whereas the prevalence of walking was negatively correlated with average air temperature (p < 0.05) (Table 1). Further explorations revealed the relationship between cycling prevalence and average distance to work to be log-linear and the relationships between cycling prevalence and average sunshine hours as well as walking prevalence and average temperature to be linear (Figure 3).

### Individual differences in cycling and walking to work

**Figure 4:**
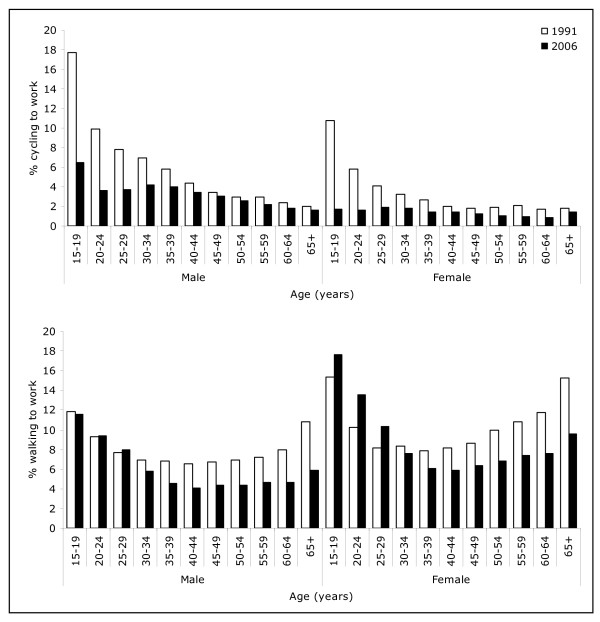
**Proportion of people who cycled and walked to work on the census day by age and gender (1991 to 2006)**.

**Figure 5:**
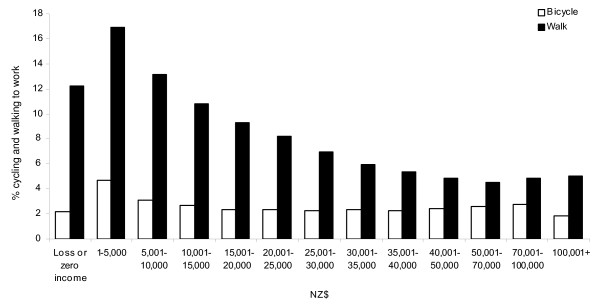
**Proportion of people who cycled and walked to work on the census day by personal income (2006)**.

Higher proportions of men compared with women cycled, while higher proportions of women walked to work (Figure 4). In 1991, the prevalence of cycling to work declined with age but this trend was less pronounced in 2006. The largest decline in cycling over the 15 year period was among younger age groups, particularly 15-19 year olds. Walking to work was least prevalent among middle-aged men and women. A significantly higher proportion of 15-29 year old women walked to work in 2006, compared with 1991. The prevalence of cycling to work did not vary significantly by personal income level whereas walking to work was less prevalent among people with higher income in 2006 (Figure 5).

## Discussion

Our analysis showed that more than four-fifths of New Zealanders used a private motor vehicle to travel to work on Census day in 2006. Only one in fourteen people walked to work and one in forty cycled. Increased car use from 1991 to 2006 occurred at the expense of active means of travel as the prevalence of using public transport remained unchanged during that period. We found important differences in active travel patterns by region, age, gender and personal income.

This is one of very few papers reporting population-based active travel behaviour in New Zealand. One of the major benefits of using Census data is that it is a near-complete survey of the general population (96.3% response rate in 2006) and the people's transport activity nationally, regionally and across different population subgroups over time may be compared. When interpreting these results, however, some limitations need to be considered. First, the Census question asked only for 'main means of travel to work' and did not take into account multiple transport modes, for example, walking and taking a bus in one journey. This means the contribution of walking to the journey to work may be under-estimated. Second, the 1991-2006 Census questions were date-specific and the data may be biased seasonally, although the timing of Census day has been similar year to year. People's active transport activity may be overestimated in this case as the Census is usually in March when the weather is warm and relatively dry. Third, we were not able to adjust for potential confounders as only aggregate data were available for this analysis. For example, personal income may be related to an individual's age, gender and residential area, all of which independently, influence choice of travel to work. Finally, the findings may be affected by the "ecological fallacy" as averaged aggregate data were used to infer relationships, for example, between various regional characteristics (such as average distance to work) and the proportion of cycling and walking to work. These questions may be addressed in future studies which obtain individual level data.

Despite these limitations, our findings are consistent with and extend the evidence gained from previous research. Parallel to decreasing trends in active travel to work behaviour, overall travel mode share for cycling and walking has been declining steadily in New Zealand (from 4% and 21% respectively in 1989 to 1% and 16% respectively in 2006) [32]. During the same period, the annual distance driven in light 4-wheeled vehicles has been increasing - particularly among the 45-64 age group [33]. From 1990 to 2006, total greenhouse gas emissions increased by 25.7%, and emissions from road transport increased disproportionately (by 66.9%) [34]. In 2006, transport accounted for 42% of total emissions from the energy sector [35]. A recent report indicates that the air quality in Auckland is worsening due to emissions from increasing use of motor vehicles [36].

A study from the US shows that CO_2 _emissions from the transport sector will continue to rise unless vehicle kilometres travelled can be substantially reduced, as present trends in car use will overwhelm the gains that may result from technological advances such as changes in fuel type (e.g., biodiesel fuel) and motor vehicle efficiency (e.g., hybrid cars) [37]. The findings are unlikely to be different in the New Zealand context given the country's dispersed population (4.3 million people spread over 268,680 km^2^), low density cities and automobile centred transportation system.

Other studies have found that New Zealanders rarely cycle or walk even when travelling short distances. Walking represents only 39% of all trips under two kilometres and cycling accounts for three percent of all trips under two kilometres and two percent of all trips between two and five kilometres in the 2004-2007 household travel surveys [38]. Only one-fifth of New Zealanders surveyed in 2003 strongly endorsed plans to replace car trips with active modes such as cycling and walking on at least two days per week and less than half of the latter considered cycling for short distances [39,40]. Although a variety of factors can influence public attitudes and behaviour [41], these findings are likely to reflect decades of under-investment in public transport and cycling and walking infrastructure. In Auckland, the construction of motorways has been favoured consistently over alternative modes in transport planning over the past 50 years [42].

We observed regional differences in patterns of cycling and walking to work. Such differences may be partly explained by aspects of the physical environment such as weather, climate and topography (hilliness) [43-45] and distance to work [46]. The influence of environmental factors such as average temperatures and rainfall, however, should not be over-emphasized. A number of cities in North America and Europe have reported substantial increases in the prevalence of walking and cycling in the last decade, for example, daily ridership doubled in New York between 2001 and 2006 [47], yet have climates much less favourable than those of most parts of New Zealand.

We found low rates of cycling to work in regions with long average distances to work (≥ 10 km). Statistics New Zealand reported that on the Census day in 2006, 83% of people who walked to work travelled less than 5 km and 89% of those who cycled to work travelled less than 10 km [26]. Although distance to work is not easily changed, increased housing density, availability of public transport and investment in active transport infrastructure such as bicycle lanes and shared paths may improve engagement in active travel modes.

Two New Zealand regions that bucked the overall trends by revealing increasing levels of walking warrant further comment. Regional strategies in Wellington and Nelson have made substantial investments in active transport. Wellington has proposed an urban development strategy [48], based on the idea of a "growth spine" (a strip of land along which more intensive urban development is encouraged), a bus lane programme [49] and school, workplace and community travel plans [50]. In Nelson, pedestrian, cycling and urban growth strategies have been implemented with integration between transport planning and urban development teams [51]. Future research will be required to investigate the effectiveness of these and other active transport strategies being implemented.

Studies from other automobile dependent countries such as the US, UK and Australia have also reported a comparatively low level of cycling and walking to work [52-56], with important sociodemographic variations in the patterns of active travel. In general, men are more likely to cycle than women; and women are more likely to walk than men. Younger people are more likely to walk and cycle compared with older age groups. This is important because it will be necessary to boost walking and cycling rates in the older age groups to realise the potential health benefits of active transport. The cardio-protective effects of exercise relate much more closely to current activity than to past exposures [57]. Our study shows that walking is more common in lower income groups, whereas socioeconomic status does not appear to influence cycling. Similar patterns were observed in previous research [53,58,59]; however, others reported associations between cycling and income [44,60].

In contrast, in most European countries, walking and cycling make up at least one-fourth of all urban trips (45% in the Netherlands) and active travel patterns are universal across different segments of society; walking increases with age, cycling declines only slightly, women cycle as much as men, and people from all income classes cycle [11,58,61-64]. The success of European countries in promoting cycling and walking is attributed to the "coordinated implementation of the multi-faceted, mutually reinforcing set of policies" in the past few decades [58,61]. Components include: provision of better facilities for pedestrians and cyclists, extensive traffic calming of residential neighbourhoods, increased traffic regulation and enforcement, people oriented urban design, integration with public transport, comprehensive traffic education and training, restrictions on car ownership, use and parking [58,61] and workplace travel plans [65].

In countries like New Zealand, significant barriers exist to implementing such comprehensive measures to promote active commuting but much could be achieved in the short term. For example, although Australia has sprawling cities and a high rate of car ownership, the prevalence of cycling to work has increased substantially in some states in the last decade together with growing investments in bicycle infrastructure (for example, there was a 43% increase from 2001 to 2006 in cycle commuters in Melbourne) [66-68]. Likewise, we found an increasing trend of walking to work in the two New Zealand regions that have invested in sustainable transport strategies.

As an important initial step at the national level, a project has begun to build a cycleway network running the length of New Zealand [69]. While primarily intended to enhance tourism, the initiative has the potential to promote active commuting if a comprehensive cycle network plan is incorporated to strengthen connections between residential areas and key activity centres in urban and rural New Zealand. A potential way to move toward more attractive environments for active commuting without major infrastructural change is reducing the speed limit in residential streets, which currently is 50 km/hr in New Zealand compared with 30 km/hr (or less) in European countries [70]. Given a favourable trend in cycling and walking as a recreational activity in New Zealand [71], another useful step would be offering interventions promoting a modal shift, i.e., from using cars to walking and cycling, tailored to recreational cyclists and walkers. The effectiveness of such targeted behaviour change programmes has been reported in a previous review [72]. An Australian study showed that participants in mass cycling events, particularly novice riders and first-time participants, cycled more frequently in the month after the event [73].

## Conclusion

Walking and cycling are increasingly marginal modes of travel to work in New Zealand and socio-demographic differences exist in such behaviour patterns. Increased walking to work, recorded in some regions, indicates that potential gains may be made with systematic promotion of active modes. Translating such successes to the national level requires political will and public support to redress decades of land use and transport policies that have prioritised car use. The rising cost of fuel linked with a resurgence in recreational cycling and walking may provide the impetus to seriously promote cycling and walking as safe and attractive choices for travel to work.

## Competing interests

The authors declare that they have no competing interests.

## Authors' contributions

STT has contributed to acquisition, analysis and interpretation of data and drafting the manuscript. AW, ST and SA have contributed to interpretation of data and revising the manuscript critically. All authors have given final approval of the version to be published.

## Authors' informations

STT - MBBS, MPH, Assistant Research Fellow, Section of Epidemiology and Biostatistics, School of Population Health, University of Auckland, New Zealand

AW - MBBS, PhD, FAFPHM, Professor and Head of School, School of Population Health, University of Auckland, New Zealand

ST - MBChB, MPH, Assistant Research Fellow, Section of Epidemiology and Biostatistics, School of Population Health, University of Auckland, New Zealand

SA - MBChB, PhD, FAFPHM, Associate Professor, Section of Epidemiology and Biostatistics, School of Population Health, University of Auckland, New Zealand

## References

[B1] Lee I, Skerett P (2001). Physical activity and all-cause mortality: what is the dose-response relation?. Med Sci Sports Exerc.

[B2] Berlin JA, Colditz GA (1990). A meta-analysis of physical activity in the prevention of coronary heart disease. Am J Epidemiol.

[B3] Colditz GA, Cannuscio CC, Frazier AL (1997). Physical activity and reduced risk of colon cancer: implications for prevention. Cancer Causes Control.

[B4] Biddle S, Biddle S, Fox K, Boutcher S (2000). Emotion, mood and physical activity. Physical activity and psychological well-being.

[B5] Ministry of Health (2003). DHB Toolkit: Physical Activity.

[B6] Haskell WL, Lee IM, Pate RR, Powell KE, Blair SN, Franklin BA, Macera CA, Heath GW, Thompson PD, Bauman A (2007). Physical Activity and Public Health: Updated Recommendation for Adults from the American College of Sports Medicine and the American Heart Association. Med Sci Sports Exerc.

[B7] Strong WB, Malina RM, Blimkie CJR, Daniels SR, Dishman RK, Gutin B, Hergenroeder AC, Must A, Nixon PA, Pivarnik JM, Rowland T, Trost S, Trudeau F (2005). Evidence Based Physical Activity for School-age Youth. J Pediatr.

[B8] Orleans CT (2000). Promoting the maintenance of health behavior change: recommendations for the next generation of research and practice. Health Psychol.

[B9] Marcus BH, Dubbert PM, Forsyth LH, McKenzie TL, Stone EJ, Dunn AL, Blair SN (2000). Physical Activity Behavior Change: Issues in Adoption and Maintenance. Health Psychol.

[B10] Hillsdon M, Thorogood M, Anstiss T, Morris J (1995). Randomised controlled trials of physical activity promotion in free living populations: a review. J Epidemiol Community Health.

[B11] Bassett DR, Pucher J, Buehler R (2008). Walking, cycling, and obesity rates in Europe, North America, and Australia. J Phys Act Health.

[B12] Hamer M, Chida Y (2008). Active commuting and cardiovascular risk: A meta-analytic review. Prev Med.

[B13] Andersen LB, Schnohr P, Schroll M, Hein HO (2000). All-cause mortality associated with physical activity during leisure time, work, sports, and cycling to work. Arch Intern Med.

[B14] Matthews CE, Jurj AL, Shu X-O, Li H-L, Yang G, Li Q, Gao Y-T, Zheng W (2007). Influence of exercise, walking, cycling, and overall nonexercise physical activity on mortality in Chinese women. Am J Epidemiol.

[B15] Appleyard D (1981). Livable Streets.

[B16] Community Livability: Helping to create attractive, safe, cohesive communities. http://www.vtpi.org/tdm/tdm97.htm.

[B17] Litman T (2007). Evaluating transportation equity: Guidance for incorporating distributional impacts in transportation planning.

[B18] Wittink R, Tolley R (2003). Planning for cycling supports road safety. Sustainable transport: Planning for walking and cycling in urban environments.

[B19] Higgins PAT (2005). Exercise-based transportation reduces oil dependence, carbon emissions and obesity. Environ Conserv.

[B20] Wu Y (2006). Overweight and obesity in China. BMJ.

[B21] The Economist (2008). Pocket World in Figures 2009 Edition.

[B22] Ministry of Transport (2008). Comparing travel modes. Household Travel Survey v1.4 revised Jan 2008.

[B23] Ministry of Transport (2002). New Zealand Transport Strategy.

[B24] Ministry of Transport (2008). The New Zealand Transport Strategy.

[B25] Transport funding realigned and increased. http://www.beehive.govt.nz/release/transport+funding+realigned+and+increased.

[B26] Statistics New Zealand (2009). Commuting Patterns in New Zealand: 1996-2006.

[B27] Badland HM, Duncan MJ, Schofield GM (2009). Using Census data to travel through time in New Zealand: patterns in journey to work data 1981-2006. New Zealand Medical Journal.

[B28] Statistics New Zealand (2002). Meshblock Database. http://www.stats.govt.nz/census/2001-census-data/2001-census-meshblock-database.aspx.

[B29] New Zealand Household travel Survey. http://www.transport.govt.nz/research/TravelSurvey/.

[B30] Statistics New Zealand Indicator 2: Living Density. http://www.stats.govt.nz/Publications/StandardOfLiving/housing-indicators/2-living-density.aspx.

[B31] NIWA National Climate Database. http://cliflo.niwa.co.nz/.

[B32] Ministry of Transport (2007). Sustainable and safe land transport: trends and indicators.

[B33] Ministry of Transport (2007). Driver travel in cars, vans, utes and SUVs. Household Travel Survey v1.2. Revised May 2007.

[B34] Ministry for the Environment (2008). New Zealand's Greenhouse Gas Inventory 1990-2006.

[B35] Ministry of Economic Development (2007). New Zealand Energy Greenhouse Gas Emissions 1990-2006.

[B36] Auckland City Council (2009). State of the environment. Update 2007/2008.

[B37] Ewing R, Bartholomew K, Winkelman S, Walters J, Chen D (2008). Growing cooler: The evidence on urban development and climate change.

[B38] O'Fallen C, Sullivan C (2009). Trends in trip chaining and tours: Analysing changes in New Zealanders' travel patterns using the ongoing New Zealand Household Travel Survey. NZ Transport Agency Research Report 373.

[B39] Badland H, Schofield G (2006). Perceptions of replacing car journeys with non-motorized travel: exploring relationships in a cross-sectional adult population sample. Prev Med.

[B40] Sullivan C, O'Fallon C (2006). Increasing cycling and walking: an analysis of readiness to change. Land Transport New Zealand Research Report 294.

[B41] Cleland B, Walton D (2004). Why don't people walk and cycle? Central Laboratories Report No: 528007.00.

[B42] Mees P, Dodson J (2006). Backtracking Auckland: Bureaucratic rationality and public preferences in transport planning. Urban Research Program Issue Paper 5.

[B43] Nankervis M (1999). The effect of weather and climate on bicycle commuting. Transp Res Part A: Policy and Practice.

[B44] Winters M, Friesen MC, Koehoorn M, Teschke K (2007). Utilitarian bicycling: a multilevel analysis of climate and personal influences. Am J Prev Med.

[B45] Parkin J, Wardman M, Page M (2008). Estimation of the determinants of bicycle mode share for the journey to work using census data. Transportation.

[B46] Badland HM, Schofield GM, Garrett N (2008). Travel behavior and objectively measured urban design variables: associations for adults traveling to work. Health Place.

[B47] Safety in numbers. http://www.transalt.org/files/newsroom/streetbeat/2009/June/0604.html#safety_in_numbers.

[B48] Wellington City Council (2006). Urban Development Strategy: Directing growth and delivering quality.

[B49] Bus Lanes. http://www.wellington.govt.nz/projects/ongoing/buslanes.html.

[B50] Regional travel plans programme. http://www.gw.govt.nz/section2271.cfm.

[B51] Strategies and Plans. http://www.nelsoncitycouncil.co.nz/strategies-plans-2/.

[B52] Plaut PO (2005). Non-motorized commuting in the US. Transp Res Part D: Transport and Environment.

[B53] Pucher J, Renne JL (2003). Socioeconomics of urban travel: Evidence from the 2001 NHTS. Transp Q.

[B54] Department for Statistics (2007). Travel to work: Personal travel fact sheet - July 2007.

[B55] Bell AC, Garrard J, Swinburn BA (2006). Active transport to work in Australia: is it all downhill from here?. Asia Pac J Public Health.

[B56] Mees P, O'Connell G, Stone J (2008). Travel to Work in Australian Capital Cities, 1976-2006. Urban Policy Res.

[B57] Lennon SL, Quindry J, Hamilton KL, French J, Staib J, Mehta JL, Powers SK (2004). Loss of exercise-induced cardioprotection after cessation of exercise. J Appl Physiol.

[B58] Pucher J, Buehler R (2008). Making cycling irresistable: lessons from the Netherlands, Denmark, and Germany. Transp Rev.

[B59] Department for Transport (2008). Transport Statistics Bulletin. National Travel Survey: 2007 Interview Data.

[B60] Moritz W (1997). Survey of North American Bicycle Commuters: Design and Aggregate Results. Transp Res Rec.

[B61] Pucher J, Dijkstra L (2003). Promoting safe walking and cycling to improve public health: lessons from The Netherlands and Germany. Am J Public Health.

[B62] Danish Ministry of Transport (2007). Danish National Travel Surveys.

[B63] Statistics Netherlands (2007). Transportation Statistics.

[B64] German Federal Ministry of Transport (2003). German Federal Travel Survey 2002 (MiD).

[B65] Land Transport New Zealand (2007). Workplace travel plan coordinator's guide.

[B66] Bauman AE, Rissel C, Garrard J, Ker I, Speidel R, Fishman E (2008). Cycling - Getting Australia Moving: Barriers, facilitators and interventions to get more Australians physically active through cycling.

[B67] Bicycle Victoria (2007). Transport and Liveability Statement provides $72 million for riders.

[B68] Rissel C (2009). Active travel: a climate change mitigation strategy with co-benefits for health. New South Wales Public Health Bulletin.

[B69] New Zealand Cycleway Project. http://www.tourism.govt.nz/Our-Work/New-Zealand-Cycleway-Project/.

[B70] Land Transport New Zealand (2003). Speed Limits New Zealand: Guidelines for setting speed limits and procedures for calculating speed limits. http://www.ltsa.govt.nz/roads/speed-limits/speed-limits-nz.html.

[B71] Sport and Recreation New Zealand (2008). Sport, recreation and physical activity participation among New Zealand adults: Key results of the 2007/08 Active NZ Survey.

[B72] Ogilvie D, Egan M, Hamilton V, Petticrew M (2004). Promoting walking and cycling as an alternative to using cars: systematic review. Br Med J.

[B73] Bowles H, Rissel C, Bauman A (2006). Mass community cycling events: Who participates and is their behaviour influenced by participation?. Int J Behav Nutr Phys Act.

